# Trans-ethnic meta-analysis identifies new loci associated with longitudinal blood pressure traits

**DOI:** 10.1038/s41598-021-83450-3

**Published:** 2021-02-18

**Authors:** Mateus H. Gouveia, Amy R. Bentley, Hampton Leonard, Karlijn A. C. Meeks, Kenneth Ekoru, Guanjie Chen, Michael A. Nalls, Eleanor M. Simonsick, Eduardo Tarazona-Santos, Maria Fernanda Lima-Costa, Adebowale Adeyemo, Daniel Shriner, Charles N. Rotimi

**Affiliations:** 1 Center for Research on Genomics and Global Health, National Human Genome Research Institute, National Institutes of Health, Bethesda, MD 20892 USA; 2 Laboratory of Neurogenetics, National Institute on Aging, National Institutes of Health, Bethesda, MD 20892 USA; 3 Data Tecnica International, Glen Echo, MD 20812 USA; 4 Longitudinal Studies Section, Translational Gerontology Branch, National Institute on Aging, Baltimore, MD USA; 5 Departamento de Genética, Ecologia e Evolução, Instituto de Ciências Biológicas, Universidade Federal de Minas Gerais, Belo Horizonte, MG 31270-901 Brazil; 6 Instituto de Pesquisa Rene Rachou, Fundação Oswaldo Cruz, Belo Horizonte, MG 30190-002 Brazil; 7 Center for Research on Genomics and Global Health, National Human Genome Research Institute, National Institutes of Health, 12 South Drive, Building 12A/Room 4047, Bethesda, MD 20814 USA

**Keywords:** Genetics, Genetic association study, Genome-wide association studies

## Abstract

Genome-wide association studies (GWAS) have identified thousands of genetic loci associated with cross-sectional blood pressure (BP) traits; however, GWAS based on longitudinal BP have been underexplored. We performed ethnic-specific and trans-ethnic GWAS meta-analysis using longitudinal and cross-sectional BP data of 33,720 individuals from five cohorts in the US and one in Brazil. In addition to identifying several known loci, we identified thirteen novel loci with nine based on longitudinal and four on cross-sectional BP traits. Most of the novel loci were ethnic- or study-specific, with the majority identified in African Americans (AA). Four of these discoveries showed additional evidence of association in independent datasets, including an intergenic variant (rs4060030, *p* = 7.3 × 10^–9^) with reported regulatory function. We observed a high correlation between the meta-analysis results for baseline and longitudinal average BP (*rho* = 0.48). BP trajectory results were more correlated with those of average BP (*rho* = 0.35) than baseline BP(*rho* = 0.18). Heritability estimates trended higher for longitudinal traits than for cross-sectional traits, providing evidence for different genetic architectures. Furthermore, the longitudinal data identified up to 20% more BP known associations than did cross-sectional data. Our analyses of longitudinal BP data in diverse ethnic groups identified novel BP loci associated with BP trajectory, indicating a need for further longitudinal GWAS on BP and other age-related traits.

## Introduction

High blood pressure (BP) is a major risk factor for cardiovascular diseases (CVD), which are leading causes of premature death worldwide^1,2^. BP has high familial clustering^3^ with heritability estimates ranging from 30 to 60%^2^, pointing to genetic association studies as valuable tools for discovering genetic determinants of BP. To date, genome-wide association studies (GWAS) have identified more than 2,000 variants significantly associated with BP (*p* < 5 × 10^–8^)^4^; however, these genetic variants explain only approximately 3.5% of BP variance^2,5^. This suggests a need to go beyond the common designs for BP GWAS, which use cross-sectional data almost exclusively and are enriched for European-ancestry individuals.

Longitudinal data recording BP trajectory have shown progressive upward trends from childhood to middle age, with some evidence of plateauing at older ages^6,7^, and, as expected, individuals who experience higher rates of BP increase are more likely to reach the elevated BP levels associated with increased risk of cardiovascular disease^8–10^. Despite this clear correlation between BP trajectory and cardiovascular-related outcomes, GWAS using empirical longitudinal BP data are currently lacking.

Longitudinal designs for BP genetic associations can identify variants associated with BP trajectory^11^ and may outperform the commonly used cross-sectional designs^12,13^. Simulations suggest that longitudinal data can more accurately identify diastolic BP loci compared to baseline data, which performs better for systolic blood pressure^13^. These findings indicate that using longitudinal BP in GWAS may enable the discovery of loci^14^ that play key roles in BP trajectory and the risk of developing CVD. Furthermore, the vast majority of BP GWAS include only or mostly individuals of European ancestry. The inclusion of multiple ethnic groups in BP GWAS has allowed the discovery of novel loci that were not detected in Europeans^1,2^.

To identify novel BP loci, we performed a series of ethnic-specific and trans-ethnic GWAS meta-analyses of longitudinal and cross-sectional data using 33,720 individuals from five longitudinal studies in the US and one in Brazil.

## Results

### Populations included and blood pressure (BP) observations

We analyzed 21,900 individuals (discovery dataset) from different ethnic backgrounds: 5304 African Americans (AA); 1439 Brazilians; 13,137 European Americans (EA); 703 Chinese Americans; and 1317 Hispanic Americans. These individuals were included from four longitudinal cohorts: (i) Atherosclerosis Risk in Communities (ARIC) Study^15^; (ii) the Bambui-Epigen Cohort Study of Aging (Bambui)^16^; (iii) the Health, Aging and Body Composition (HABC) Study^17^ and (iv) the Multi-Ethnic Study of Atherosclerosis (MESA)^18^. The studied individuals showed ancestry profiles consistent with expectations given their self-reported ethnic backgrounds (Fig. S1). Interestingly, the average diastolic BP (DBP) in the Bambui-Brazil Aging Cohort is 83.41 mm Hg (SD = 12.72), similar to the average DBP in the Brazilian general population of similar age^19^, but considerably higher than the average in the HABC study, also a study of older adults (71.30, SD = 11.76), and higher by ~ 10 mm Hg compared to the other analyzed groups (Table 1). We observed higher levels of BP and hypertension in African Americans (AA) compared with other ethnicities (Table 1), which is in agreement with previous reports^20^. However, for the older individuals (Bambui-Brazil and HABC), the mean BP and prevalence of hypertension (> 65%) were similar even among different ethnicities. Furthermore, the longitudinal data showed that increasing baseline age is correlated with a lower slope of systolic and diastolic blood pressure (SBP and DBP) in European- and African-ancestry populations (Fig. S2), consistent with previous studies^21,22^. Most of the baseline and follow-up ages in the cohorts included in our meta-analysis were after the expected inflection point in DBP (Table 1), as shown in Fig. S2B. Consistently, after adjustment for age^2^ (our study model) or age + age^2^, as separate covariates, there was no difference between DBP trajectory estimates (Fig. S2D).

**Table 1:** Study participants' baseline characteristics in the discovery and replication datasets.

Study	Ethnicity^1^	N	Age (years)	BMI (kg/m^2^)	Systolic BP (mmHg)	Diastolic BP (mmHg)	Medication (%)	Hypertension (%)^2^
**Discovery data**								
ARIC	AA	2764	53.49 (5.81)	29.72 (5.99)	128.56 (20.90)	79.91 (12.10)	1236 (45%)	1633 (60%)
	EA	9170	54.36 (5.68)	27.00 (4.80)	118.48 (16.86)	71.64 (9.95)	2333 (25%)	2951 (32%)
Bambui	Brazilians	1439	69.09 (7.19)	25.14(4.97)	137.34 (22.54)	83.41 (12.72)	735 (51%)	1109 (77%)
HABC	AA	1115	73.43 (2.89)	28.54 (5.35)	138.82 (22.44)	73.42 (12.70)	699 (63%)	860 (77%)
	EA	1641	73.77 (2.84)	26.63 (4.13)	133.55 (20.11)	69.86 (11.13)	816 (50%)	1067 (65%)
MESA	AA	1425	62.00 (10.07)	30.23 (5.90)	131.50 (21.42)	74.48 (10.15)	684 (48%)	849 (60%)
	EA	2326	62.66 (10.27)	27.83 (5.05)	123.67 (20.46)	70.20 (9.92)	626 (27%)	895 (38%)
	Chinese	703	62.61 (10.42)	23.96 (3.33)	124.01 (21.43)	71.82 (10.36)	195 (28%)	268 (38%)
	Hispanics	1317	61.48 (10.38)	29.45 (5.16)	126.83 (22.12)	71.49 (10.08)	389 (30%)	548 (42%)
Total		21,900						
**Replication data**								
WHI	AA	6500	61.14(6.86)	31.04(6.29)	131.40(17.84)	78.02(9.48)	2982(46%)	3954 (61%)
	Hispanics	2869	60.55(6.55)	28.81(5.42)	125.08(16.94)	74.83(9.48)	601(21%)	995 (35%)
Framingham	EA	2451	55.39(9.39)	27.43(4.16)	126.10(18.65)	74.57(9.84)	396(16%)	763(31%)
Total		11,820						
Total		33,720						

^1^AA = African Americans and EA = European Americans.

^2^Hypertension was defined as SBP at least 140 mmHg, DBP at least 90 mmHg, or being on antihypertensive medication.

Mean (SD) reported, except where indicated.

### Meta-analysis of longitudinal and cross-sectional data

We performed GWAS by ethnic group and cohort, followed by ethnic-specific and trans-ethnic meta-analysis for each longitudinal BP outcome: the trajectories (i.e. rate of change of BP per year) of (i) systolic and (ii) diastolic BP, and the averages (i.e*.* mean of longitudinal BP measurements) of (iii) systolic and (iv) diastolic BP. To facilitate comparisons between the longitudinal BP analyses and analyses conducted using a more standard cross-sectional approach, we also performed meta-analysis for cross-sectional SBP and DBP at baseline using the individuals included in the longitudinal analyses. We present associations from the discovery data that reached the standard genome-wide statistical significance (p < 5 × 10^–8^) and borderline genome-wide statistical significance (p < 9 × 10^–8^). For the statistically significant associations, we present the statistical significance for the three outcomes analyzed: trajectory, average, and baseline (Table 2 and Table S1).

**Table 2:** Genome-wide statistically significant (*p* < 5 × 10–8) and borderline genome-wide statistically significant (*p* < 9 × 10–8) associations in the GWAS meta-analysis which had additional evidence of association in independent datasets.

Index variant (gene)	B37 Chr:Pos (consequence)	1000 Genomes freq	Tested	Ethnicity^1^	Trait^2^	N	Effect^3^	SE	Trajectory	Average	Baseline
		AFR/AMR/EAS/EUR	MAF						P-value	P-value	P-value
rs565370904* (*LRP12*)	8:105526773 (intronic)	delA: 0.38/0.3/0.38/0.35	0.337	EA	SBP_Trajectory	11,413	0.088	0.016	**3.93E-08**	6.15E−06	1.10E−04
rs199848402* (*HCN1*)	5:45940445 (intergenic)	insTA: N/A	0.387	EA	DBP_Trajectory	11,413	0.078	0.014	**4.29E-08**	0.02	0.71
rs4060030* *C8orf37-AS1*)	8:96442763 (intronic)	C: 0.41/0.71/0.78/0.74	0.494	AA	DBP_Trajectory	4091	− 0.125	0.023	**5.97E-08**	2.25E−04	0.05
rs74838709* (*GABRG3*)	15:27787222 (Intergenic)	A:0.00/0.01/0.00/0.04	0.013	AA	SBP_Baseline	2540	16.482	3.006	0.91	4.0E−04	**4.18E−08**

*Novel blood pressure associations (250 kb away from known loci).

*N/A* Not Available

^1^AA = African American, EA = European-American, TRANS = Transethnic.

^2^SBP = Systolic blood pressure, DBP = Diastolic blood pressure.

^3^The variant effect corresponds to the outcome that was statistically significant.

**Figure 1:**
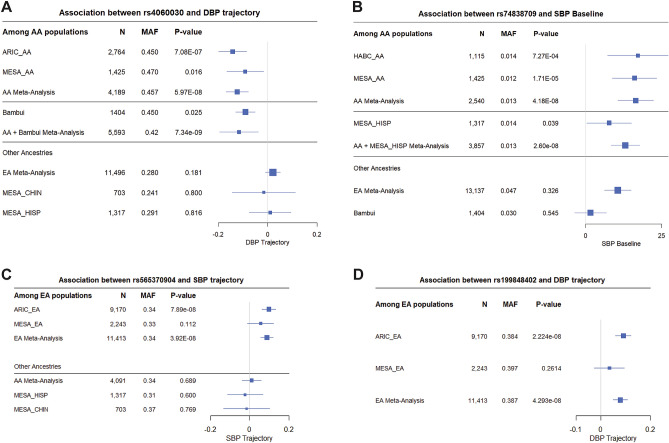
Forest plots of the genome-wide statistically significant associations of longitudinal and baseline BP data with additional evidence of association in independent datasets. Forest plots show β values (95% confidence intervals) and *P*-values from the linear regression of longitudinal outcomes (BP trajectory or BP average) adjusted for age^2^, sex, BMI, principal components (PC1 and PC2), and the use of antihypertensive medications. Populations not shown in the plots did not have the genetic variant to perform association analyses due to low allele frequency or because the variant was pruned during quality control.

**Figure 2:**
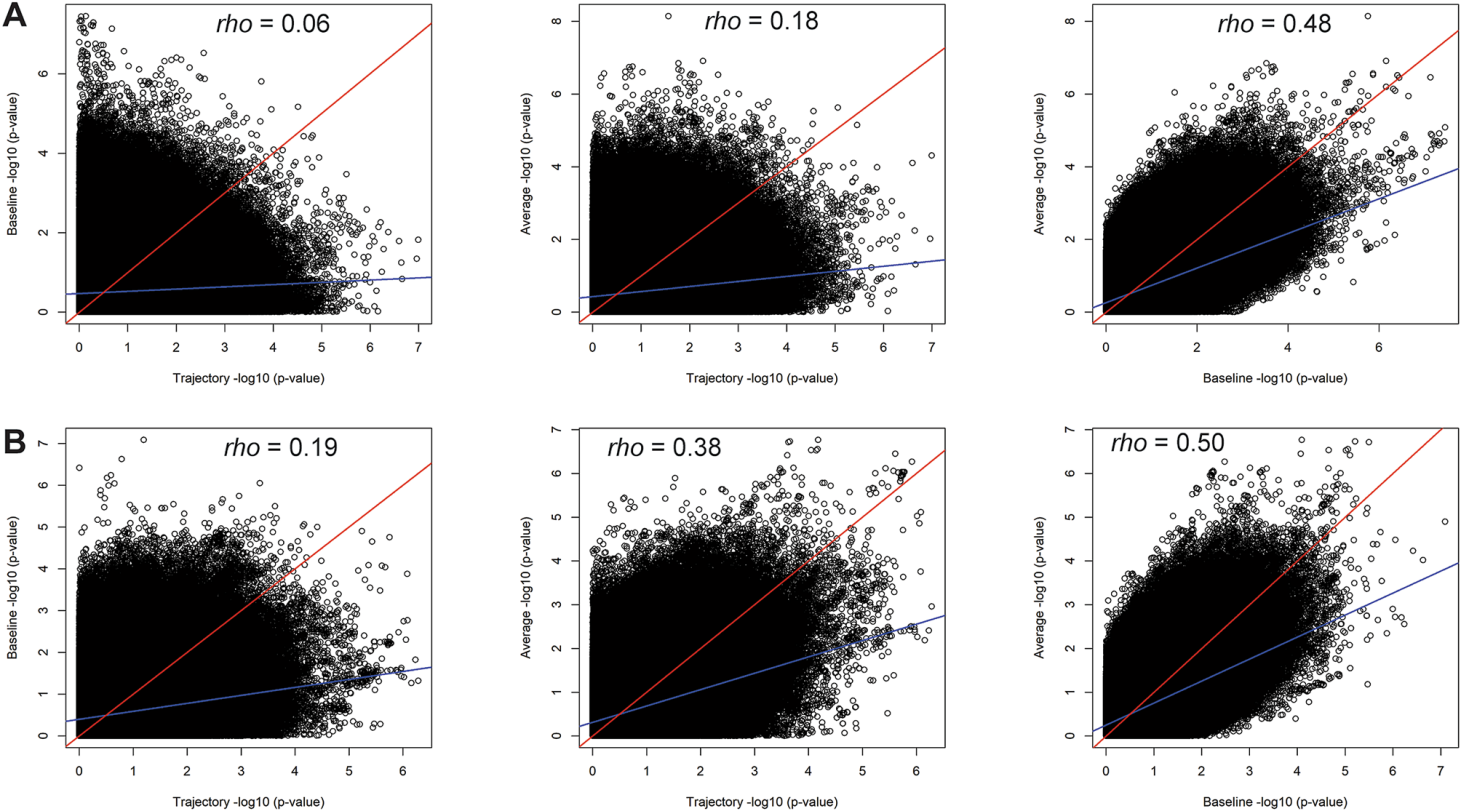
Comparison between longitudinal and cross-sectional SBP data. Correlation between the meta-analysis results using (**A**) African American and (**B**) European American data. We combined 5293 African Americans and 13,120 European Americans from the ARIC, MESA, and HABC studies (Table 1). We used ~ 20 million variants tested for association among African Americans and ~ 10 million variants for European-Americans. The blue lines represent the fitted linear regressions and the red lines represent the x = y line. All correlations had *p* ≤ 2.2 × 10^−16^.

We found 19 loci that were significantly (or borderline) associated with BP outcomes (Table 2, Tables S1–S4, Fig. 1 and Figs. S3–S10), of which 14 were significantly associated and five were borderline significantly associated. Out of these 19 loci, 10 were associated with longitudinal outcomes and nine with baseline. Of these 19 loci, 17 were ethnic- or study-specific, with the majority (11) found in African Americans (AA). We observed significant heterogeneity across studies (ethnic-specific meta-analyses) or ethnicities (trans-ethnic meta-analyses) for only one locus in AA populations (Table S2). This variant (rs145522449) showed significant heterogeneity in effect sizes in MESA and ARIC even though the direction of effect was the same in both studies. Interestingly, 9 out of 10 BP loci detected using longitudinal outcomes were novel, whereas only 4 of 9 BP loci using the cross-sectional outcomes were novel (Tables 2 and S1). This suggests that longitudinal data allow for discovery of novel associations that could not be identified using baseline data alone. All signals based on average BP also had some evidence of association (*p* < 0.05) in the cross-sectional analyses, consistent with the fact that the cross-sectional data are included in the calculation of average BP. This trend of overlapping associations was not observed for BP trajectory (Tables 2 and S1). To evaluate the overall correlation between the ability of longitudinal and cross-sectional data to identify BP associations, we compared the statistical significance of all tested genetic variants (Fig. 2). We identified at least twofold higher correlation between average and cross-sectional findings (*rho* = 0.48 in African Americans, Fig. 2A, and *rho* = 0.50 in European Americans, Fig. 2B) compared to the correlation between trajectory and cross-sectional findings (*rho* = 0.06 in African Americans, Fig. 2A, and *rho* = 0.19 in European Americans, Fig. 2B). Consistently, we observed the highest correlation between the results using baseline and average BP outcomes, and trajectory was more correlated with average than with baseline outcomes (Fig. S11).

### Discovery findings with additional evidence of association

Out of the 19 variants with a GWAS statistically significant association (or borderline) in the discovery analysis, 10, 11, and eight variants were available (passed frequency and QC criteria) in the EA, AA, and Latinos/Brazilians datasets, respectively (Fig. 1 and Table S1). We identified four variants with additional evidence of association with BP in two replication datasets (same direction and p-value < 0.05, Tables 2 and S3) and we did not identify statistically significant local replications. While some discovery associations did not show additional evidence of association in the replication datasets (Table S3), in some cases because of the absence of the variant in the replication dataset due to filtering for minor allele frequency and quality control procedures, these were still described (SI text) as promising variants based on our comprehensive annotation (Table S4).

The C allele of an intronic variant (rs4060030) in the RNA gene *C8orf37-AS1* was associated (β = − 0.125, SE = 0.023, *p* = 5.9 × 10^–8^) with decreasing DBP trajectory in AA populations (Fig. 1A, Table 2, Tables S1-S4, and Fig. S8). This association was replicated using longitudinal BP data of Brazilians (Bambui-Brazil) (*p* = 7.3 × 10^–9^, after meta-analysis including AA and Brazilians). This variant was also associated in AA with decreasing SBP trajectory (β = − 0.102, SE = 0.023, *p* = 7.1 × 10^–5^) and decreasing SBP average (β = − 0.748, SE = 0.203, *p* = 2.2 × 10^–4^), and associated with decreasing SBP average (β = − 0.512 mm Hg, SE = 0.243, *p* = 0.03) in Hispanic women from the Women's Health Initiative (WHI). The rs4060030 variant is involved in epigenetic regulation in several tissues, including cardiac, and differentially binds 14 regulatory motifs, including YY1, for which expression is increased in hypertensive elastic arteries^23^. Furthermore, this variant is a statistically significant *eQTL* of an uncharacterized gene (LOC101927039) in the heart atrial appendage^24^.

The SNP rs74838709 was genome-wide associated with baseline SBP (β = 16.5 mm Hg , SE = 3.0, *p* = 4.2 × 10^–8^) in the AA meta-analysis with replication observed in Hispanics from the WHI study (*p* = 1 × 10^–8^, after meta-analysis including AA and Hispanics) (Fig. 1B, Table 2, Tables S1-S4, and Fig. S7). rs74838709 is an intergenic variant located ~ 7 kb downstream of the *GABRG3* gene, which has been reported associated with several traits including coronary artery calcification^25^, cognition^26^, and weight measures^27^. This variant has a regulatory motif altered in the ZEB1 transcription factor (*ZEB1* is located in an “obesity” locus^28^), which regulates the accumulation of adipose tissue^29^*.*

An intronic deletion rs565370904 was associated with SBP trajectory in EA populations (β = 0.088, SE = 0.016, *p* = 3.9 × 10^–8^) (Fig. 1C, Table 2, Tables S1-S4). The rs565370904 variant was associated with cross-sectional SBP in the Hispanics from WHI (*p* = 0.03) and was borderline in the UK BioBank (*p* = 0.051; Table S3. This deletion is in the gene *LRP12* (LDL Receptor Related Protein 12), which has low-density lipoprotein particle receptor activity^30^.

We also identified an intergenic insertion (rs199848402) associated with DBP trajectory in EA (β = 0.079, SE = 0.014, *p* = 4.3 × 10^–8^) (Fig. 1D, Table 2, Tables S1-S4). rs199848402 was associated with cross-sectional DBP in AADM (p = 0.045) and was borderline in the Framingham study (p = 0.06) (Table S3). rs199848402 is in a genomic region downstream of *HCN1*, previously associated with electrocardiographic P Wave duration in European-ancestry individuals^31^.

### Meta-analyses of discovery and replication studies

In addition to our discovery and replication design, we also conducted meta-analyses of discovery and replication studies together. All statistically significant and suggestive results (p < 1 × 10^–6^) are shown in Table S5. Notably, the trans-ethnic meta-analysis of average BP outcomes, including all studied individuals, identified (p < 5 × 10^–8^) two common frequency loci, represented by the variants rs6599176 and rs11105368 in the genes *ULK4* and *ATB2B1*, respectively. These two loci have been consistently reported to be associated with BP and hypertension^20,32^.

### SNP heritability of the longitudinal and cross-sectional BP traits

To calculate SNP heritability (*h*^2^), we used European American (EA) individuals from the ARIC and MESA studies. Regardless of antihypertensive medication use, DBP outcomes tended to have higher *h*^2^ than SBP outcomes (Table 3). Notably, the *h*^2^ of average DBP (*h*^2^ = 0.47, SE = 0.04) was higher than the *h*^2^ of baseline DBP (*h*^2^ = 0.28, SE = 0.04). The use of antihypertensive medication appeared to systematically decrease *h*^2^. We did not evaluate *h*^2^ in AA individuals due to limited sample size.

**Table 3:** Heritability analyses in European Americans using longitudinal and cross-sectional BP outcomes.

BP outcome	Heritability (h^2^)	SE	p-value
**European Americans**^**1**^			
SBP trajectory	0.2621	0.0445	5.31E−11
SBP average	0.2684	0.0443	1.15E−11
SBP baseline	0.2002	0.0449	1.06E−06
DBP trajectory	0.3712	0.0421	6.38E−19
DBP average	0.4656	0.0412	5.83E−30
DBP baseline	0.2795	0.0428	1.23E−14
**European Americans (not using antihypertensive medications)**^**2**^			
SBP trajectory	0.3152	0.0590	2.34E−09
SBP average	0.3638	0.0587	5.75E−12
SBP baseline	0.2625	0.0599	1.56E−06
DBP trajectory	0.4583	0.0560	1.42E−16
DBP average	0.5698	0.0547	1.05E−25
DBP baseline	0.3315	0.0565	3.82E−12

^1^The "European Americans" heritability analysis was performed using 389,360 SNPs and 8,515 European Americans from the ARIC and MESA studies.

^2^The "European Americans (not using antihypertensive medications)" heritability analysis was performed using 389,360 SNPs and 6,372 European Americans from the ARIC study.

### Identifying previous GWAS BP associations in longitudinal and cross-sectional BP datasets

The power of longitudinal compared to cross-sectional data to identify known BP loci was evaluated in GWAS Catalog associated BP traits^4^ (Methods). From those associations with reported risk alleles, 1957 variants (all associated with cross-sectional BP traits) were present in our combined data and 317 associations had the risk allele associated in the same direction reported in the GWAS Catalog (Fig. S12 and Tables S7 and S8). Consistent with the level of correlation between longitudinal and baseline results (Fig. 2), for both SBP and DBP, the highest number of shared identifications (associations using different BP outcomes) were found using average and the baseline (85), followed by average and trajectory (40), and then trajectory and baseline (25) (Fig. S12). The longitudinal outcomes identified similar numbers of previous associations: 185 using average and 177 using trajectory, but more (20% and 15%) than baseline (154). Considering the exclusive identifications (*i.e.*, identified by only one outcome), the highest number (132) was identified by the trajectory, followed by average (80) and baseline (64). These results suggest that the BP longitudinal outcomes may have greater power to identify variants associated with BP.

### Sensitivity analyses

We performed three additional analyses to assess sensitivity to assumptions for the five variants discovered using BP trajectories. First, adjusting for the top five principal components, instead of the top two principal components, did not change the estimates of either effect sizes or standard errors (Table S6). This analysis is consistent with a lack of confounding by weaker components of population structure. Second, adjusting trajectories for baseline BP values led to small reductions in both effect sizes and standard errors, resulting in minor increases in *p*-values (Table S6), as expected given correlation between trajectories and baseline values. This analysis indicates that trajectories are capturing substantive information beyond baseline values. Third, adjusting for the BMI trajectory did not change effect sizes or standard errors (Table S6), consistent with a lack of confounding between BMI and BP trajectories.

## Discussion

We report the first GWAS meta-analysis using longitudinal BP data from diverse populations. By performing ethnic-specific and trans-ethnic meta-analyses, we showed that: (i) longitudinal data identified novel BP loci not identified using cross-sectional data; (ii) despite the correlation between longitudinal and cross-sectional BP outcomes, there is evidence for different genetic associations underlying these traits; (iii) longitudinal BP outcomes identified more of the previously published BP associations than cross-sectional outcomes identified; (iv) as was found with cross-sectional associations, most of the associations identified using longitudinal outcomes were ethnic-specific; and (v) longitudinal BP traits had higher heritability than baseline BP traits.

Our findings indicate that longitudinal data allows for the identification of novel BP associations as well as replication of known BP loci, suggesting a higher statistical power of longitudinal data for identifying GWAS BP loci, in agreement with a previous report^13^. We identified three novel BP trajectory associations with additional evidence of association in replication datasets (Table 2) with likely biological implications. Notably, one variant (rs4060030) in the RNA gene *C8orf37-AS1* was associated with decreasing DBP trajectory in AA populations and Brazilians (Fig. 1A and Fig. S8, Table 2, Tables S1-S4). Interestingly, rs4060030 was also associated with decreasing SBP trajectory in AA and Hispanic women, suggesting that this locus affects both DBP and SBP trajectory. This variant differentially binds 14 regulatory motifs, including YY1, for which expression is increased in hypertensive elastic arteries^23^. This variant is a statistically significant eQTL of *LOC101927039* in the heart atrial appendage^24^. *C8orf37-AS1* has been reported to be associated with heart rate response to beta-blockers^33^.

As expected, we observed correlation between the meta-analyses results using longitudinal and cross-sectional BP data, with the cross-sectional results more correlated with average than trajectory results. Although most of the statistically significant associations using longitudinal data showed some evidence of association (*p*-value < 0.05) using cross-sectional data, they did not reach genome-wide statistical significance (Table S1). In contrast, two variants (rs199848402 and rs140355897) that were significantly associated with DBP trajectory showed no evidence of association using the cross-sectional data (*p*-values = 0.71 and 0.77, respectively). These results indicate that novel variants identified by the longitudinal analysis could not be identified using the standard cross-sectional design alone and that, despite some degree of overlap between longitudinal and cross-sectional data, there is evidence for different genetic associations underlying these traits. Genetic variants associated with BP trajectory but not with cross-sectional measurements may be of particular significance, as understanding the underlying biological mechanisms could give us new insight into causes of steeper declines in BP over time and could potentially lead to new therapies for hypertension prevention. Furthermore, our results warrant new studies to address the question of whether BP trajectory-associated variants, singly or in aggregate, are also associated with the development of cardiovascular diseases such as myocardial infarction and stroke.

Among the 19 loci that were significantly or borderline associated with BP outcomes, 11 were identified in African-ancestry individuals (Table S1 and S7), despite smaller sample sizes compared with European-ancestry populations in the discovery analysis. This is in accordance with the higher than expected contribution of AA to genome-wide statistically significant associations compared with those of European or Asian ancestry given the proportion of GWAS that include those of African ancestry^34^. These results highlight the importance of including diverse ethnic populations to identify associations for variants absent or with very low frequency in European populations.

The *h*^2^ of average DBP was higher than the *h*^2^ of cross-sectional DBP in EA populations, suggesting that it may be easier to identify genetic determinants of DBP using average compared to cross-sectional DBP data in EA (Table 3), in agreement with previous reports^13^. Higher heritability after excluding those on antihypertensive medications was observed in EA (Table 3), but limited sample size in AA precluded this analysis among AA. Considering the ethnic-specific nature of BP heritability^35^, similar analyses should be performed to compare longitudinal and cross-sectional BP outcomes using AA and other ethnic groups.

Although most of the individuals (65%) included in our discovery data were not using antihypertensive medications, we recognize their potential effect on modeling BP trajectory. The effects of BP-lowering treatment and regimen adherence and compliance on BP outcomes are problematic, not only for longitudinal studies but also for cross-sectional BP studies^36^. To model the effect of the use of antihypertensive medication on the BP trajectories, visit-specific information on medication use was incorporated into the mixed model analysis.

DBP rises essentially linearly until around the sixth decade of life and then tends to flatten or decline^37^. Furthermore, our longitudinal data showed that BP trajectories show more non-linear (quadratic) behavior on an individual basis (Fig. S2B) than at the population level (Fig. S2C). We adjusted for age^2^ to account for this tendency. However, given that most of the baseline and follow-up ages were after the expected inflection point in DBP (Fig. S2B and Table 1), our DBP trajectories estimated in older individuals do not capture the trajectory in early life, prior to the inflection point.

In conclusion, these results show that GWAS and meta-analysis using longitudinal data enable the discovery of novel genetic variants that may have an important role in disease progression and age-related traits. More longitudinal data may facilitate the discovery of variants associated with the progression of hypertension, as well as other traits highly affected by age, such as type 2 diabetes, chronic kidney disease, metabolic syndrome, body mass index, and cognition.

## Methods

### Discovery datasets: study participants and longitudinal blood pressure data

Four studies were included in the discovery analyses: (i) the Atherosclerosis Risk in Communities (ARIC) Study^15^; (ii) the Bambui-Epigen Cohort Study of Aging (Bambui)^16^; (iii) the Health, Aging and Body Composition (HABC) Study^17^ and (iv) the Multi-Ethnic Study of Atherosclerosis (MESA)^18^.

The ARIC study^15^ is a prospective cohort study of atherosclerosis (dbGaP Study Accession: phs000090.v1.p1) including 15,792 African Americans (AA) and European Americans (EA) aged 45–64 at baseline, residing in four communities in the United States (Forsyth County, NC; Jackson, MS; the northwest suburbs of Minneapolis, MN; and Washington County, MD). The ARIC study participants were examined up to four times, with visits occurring in 1987–1989 (baseline), 1990–1992, 1993–1995, and 1996–1998.

The Bambui study^38^ is a population-based cohort study of aging in the city of Bambui in Southeast Brazil. The population eligible for the cohort comprised all residents aged 60 years and over on 1 January 1997 (1,742 inhabitants) and 92% of the eligible population participated in the study^16^. BP was measured at baseline (1997) and in three subsequent waves (2000, 2002, and 2008)^38^. This cohort is composed of admixed individuals with European (78.5%), African (14.7%), and Amerindian (6.7%) ancestries^39^.

HABC^17^ is a cohort study of aging (dbGaP Study Accession: phs000169.v1.p1) comprising AA and EA (3,075 men and women, aged 70–79 at baseline) who were recruited in 1997–1998 and had annual clinical follow-up for six years.

MESA^18^ is a prospective study of atherosclerosis (dbGaP Study Accession: phs000209.v13.p3) involving a population-based sample of 6,814 asymptomatic men and women aged 45–84. Participants included African Americans, Hispanic Americans, Chinese Americans, and European Americans from six field centers in the United States (Wake Forest University, Columbia University, Johns Hopkins University, University of Minnesota, Northwestern University, and University of California, Los Angeles). The first examination (baseline) took place from July 2000 to July 2002 and was followed by three examination periods that were 17–20 months in length.

### Replication datasets: study participants and longitudinal blood pressure data

We used two additional longitudinal datasets to perform exact and local^40^ replication analysis: the Framingham Heart Study (FHS) (dbGaP Study Accession: phs000007.v30.p11) and the Women's Health Initiative (WHI) (dbGaP Study Accession: phs000200.v1.p1). Exact replication is when the replication dataset replicates (same direction and p-value < 0.05) the same (“exact”) variant and trait identified during the discovery analysis. Local replication accounts for possible differences in LD patterns between studies and therefore interrogates a set of variants in LD (*r*^2^ > 0.3 in the discovery dataset) with the exact variant. The p-values for the associations of these correlated variants are adjusted for the effective number of tests, following the example in^40^. In addition, we performed in silico replication by looking up the BP results (summary statistics) in public cross-sectional studies: UK BioBank^41^, The Uganda Genome Resource^42^ and The Africa America Diabetes Mellitus study^43^.

### Genotyping and quality control

We used the genotype data (Illumina Infinium Omni2.5) from 1442 participants from the Bambui Study in the context of the EPIGEN project^39^. For the ARIC and MESA studies, we used the genotype data (Affymetrix Genome-Wide Human SNP Array 6.0) from 12,773 and 6814 participants, respectively. For the HABC study, we used the genotype data (Illumina Human1M-Duo) from 2870 individuals. We split all genotype study datasets by ethnicity (i.e., African Americans, European Americans, Hispanic Americans, and Chinese Americans) before quality control, phasing, imputation, and downstream analyses. The Brazilian Bambui Study could not be meaningfully subdivided by ethnicity because this population is highly admixed^39^.

For all genotype datasets, we applied standard data quality control and imputation procedures. Quality control was performed using PLINK software^44^ by applying the following filters: minor allele frequency (–maf 0.01), missing rate per variant (–geno 0.05), missing rate per individual (–mind 0.05), and Hardy–Weinberg equilibrium (–hwe 0.000001).

### Population structure and relatedness

Before inference of population structure and relatedness, we pruned strand-ambiguous SNPs and SNPs in linkage disequilibrium (r^2^ > 0.2, PLINK filter—indep-pairwise 50 10 0.2). Considering that the datasets included in this study have multiple ethnicities, we performed principal component analysis (PCA) to assess population structure. We merged each study genotype dataset with the 1000 Genomes Project Phase 3 data^45^ and performed PCA using EIGENSTRAT^46^. To infer the level of relatedness among individuals, we calculated the pairwise kinship matrix for each dataset using EPACTS^47^.

### Phasing and imputation

To generate valid VCF files before imputation and association tests, we used the tool checkVCF.py (https://genome.sph.umich.edu/wiki/CheckVCF.py) and bcftools^48^ to check and correct for monomorphic sites, consistency of reference alleles with the reference genome, variants with invalid genotypes, and non-SNP sites.

For the ARIC, Bambui, and MESA studies, we used the Sanger server (https://imputation.sanger.ac.uk/) for phasing and imputation of the genotype data, using the EAGLE2^49^ and PBWT^50^ software, respectively. For Chinese, European, and Hispanic Americans, we used the 1000 Genomes Phase 3 reference panel, and for African Americans and Brazilians, we used the African Genome Resources panel^51^. For the HABC genotype data, we used the Michigan Imputation Server (https://imputationserver.sph.umich.edu/) with EAGLE2 phasing^49^. The HABC African Americans were imputed with the CAAPA African American reference panel and the European Americans were imputed using the Haplotype Reference Consortium (HRC r1.1 2016) panel.

### Assessing blood pressure longitudinal measurements

We used two different longitudinal blood pressure (BP) outcomes, trajectory and average, obtained for systolic and diastolic BP (SBP and DBP) measurements across consecutive exams of each participant. To reduce the bias known as the white coat syndrome^52^, we calculated the average of the second and third measurements (discarding the first) to obtain the SBP and DBP measurements for each exam. For the exams with no data for the third BP measurement, we included only the second measurement. All the BP outcomes were obtained or calculated by study and ethnic group.

To assess the BP trajectory outcome, we followed the methodology used in Gouveia et al.^53^ to assess cognitive trajectory in Brazilians. We used the linear mixed models function implemented in the R package lme4^54^ to fit and assess the per-individual systolic and diastolic BP trajectories (*i.e.*, rate of change per year) for individuals with at least two BP measurements during follow-up. Specifically, we fitted the mixed effects model (1) below for individual j at visit i, where X_k_ (k = 1, 2, 3, …, n) are n covariates with fixed-effects β_k_ (k = 1, 2, 3, …,n).

1 $$Y_{ij} = \, \left( {\beta_{00} + u_{0j} } \right) \, + \, \left( {\beta_{1} + u_{1j} } \right) \, Age_{ij} + \, \Sigma_{k} \beta_{k} X_{kij} + \, e_{ij} ,\quad {\text{where}}\quad k = 1,2,3, \ldots , \, n; \, u_{0j} , \, u_{1j} \sim \, iid$$

where u_0j_ is a random variable that allows variation around the intercept β_00_ for each individual j, u_1j_ allows for variation around the coefficient β_1_, and e_ij_ is the subject-level residual. In line with standard practice, we assumed each of the random effects to be independent and normally distributed with unstructured correlation between u_0j_ and u_1j_. We then used the predicted random effect u_1j_ from model (1) as the phenotype for GWAS. Models were adjusted for age^2^, sex, BMI, use of antihypertensive drugs as a binary covariate, and population structure using the two first principal components (PCs). We used visit-specific covariates for those (age^2^, BMI, and use of antihypertensives) that may vary over time.

The BP average outcome was obtained by calculating the average of blood pressure measurements (SBP and DBP) over time. Then, we used the BP longitudinal trajectory and average values as outcomes for our analyses. To compare the longitudinal BP outcomes with cross-sectional BP outcomes, we also performed the analyses using the baseline BP measurements as a cross-sectional outcome using the same set of individuals included in the longitudinal analysis (Table 1).

### GWAS and meta-analysis

We performed genome-wide association studies (GWAS) by cohort and by ethnic group (Table 1) followed by ethnic-specific meta-analysis and trans-ethnic meta-analysis (including all discovery datasets). Association analyses were performed for longitudinal (trajectory and average) and baseline BP outcomes, using linear mixed models as implemented in EPACTS^47^. For cross-sectional and average BP, models were adjusted for baseline age^2^, sex, BMI (except for average BP), use of antihypertensive drugs, and population structure using the two first principal components (PCs). We used average BMI in the average BP models. To avoid over-adjustment, models previously adjusted during the estimation of BP trajectory (see previous section) were not adjusted in the association analysis. To account for relatedness among samples, kinship matrices were incorporated as random effects in association analyses. Markers with minor allele frequency > 0.01 and imputation info score > 0.3 were retained for the GWAS analyses. We used genotype dosages instead of hard genotype calls to allow for the uncertainty of the imputed calls to be considered within the association model. From each GWAS results, we extracted summary statistics (standard errors, sample size, and direction of effect) to perform fixed-effect meta-analysis weighted by sample size of each study using METAL^55^. We set the mode HETEROGENEITY to determine whether observed effect sizes (or test statistics) were homogeneous across samples. Genome-wide statistical significance for the meta-analysis was set at a *p*-value of 5 × 10^–8^ and borderline statistical significance was set at a *p*-value of < 9 × 10^–8^. We considered an association from meta-analysis to be novel if no previous BP genome-wide associations with cross-sectional BP were reported in the GWAS Catalog (reference file “All associations v1.0.2”) within ± 250 kb of our detected association.

### Annotation

For our comprehensive annotation of the statistically significant associations, we utilized a series of sources: dbSNP^56^, Ensembl^24^, the human genome browser at UCSC^57^, GTEx Portal release V7^58^, HaploReg v4.1^59^, GWAS catalog^4^, PANTHER^60^, STRING^61^, International Mouse Phenotyping Consortium (IMPC)^62^ and Rat Genome Database^63^.

### Identification of previous BP associations from the GWAS catalog

To test if our ethnic-specific and trans-ethnic meta-analysis approaches using longitudinal and cross-sectional data identify previously published associations with BP, we used the GWAS Catalog^4^ (reference file “All associations v1.0.2”, downloaded 08/13/2019). From this database, we considered only the statistically significant associations (*p* < 5 × 10^–8^) for which the risk allele was reported, with the following disease/trait terms: (i) “blood pressure”, (ii) “systolic blood pressure”, (iii) “diastolic blood pressure”, (iv) “systolic blood pressure change trajectories”, or (v) “pulse pressure”. Then, we evaluated 1957 GWAS Catalog associations for association using our meta-analysis data. We considered identification successful for associations with p < 0.05 for the same risk allele and the same direction of effect as reported in the GWAS Catalog.

### Heritability analysis

For the heritability analysis, we used the European Americans (EA) and African Americans (AA) from the ARIC and MESA studies, since these samples have similar BP characteristics (Table 1). The genotype data were merged by ethnic group followed by pruning SNPs with missing data > 0.05 (PLINK filter –geno 0.05) and the SNPs in high linkage disequilibrium (r^2^ > 0.8, PLINK filter –indep-pairwise 50 10 0.8). We used the GCTA-GREML method^64^ to estimate the heritability of the longitudinal and cross-sectional BP outcomes adjusted for age^2^, sex, BMI, medication, and study. Because this method assumes unrelated individuals, we removed one of a pair of individuals with estimated relatedness > 0.2, maximizing the samples remaining for analysis^64^.

### Ethics statement

All dbGaP studies (dbGaP Study Accession described in the Methods section) obtained ethical approval from the relevant institutions and written informed consent from each participant prior to participation. We obtained approval for controlled access (protocol number: 12-HG-N185) of each of the dbGaP studies. Brazil’s national research ethics committee approved genotyping as part of the Epigen-Brazil protocol (Brazilian National Ethics Research Council (CONEP), resolution 15,895). All methods were performed in accordance with the CONEP guidelines and regulations.

## Supplementary information

Supplementary Information 1.

Supplementary Information 2.

Supplementary Information 3.

Supplementary Information 4.

Supplementary Information 5.
