# Supplementary material for: Trans-ethnic meta-analysis identifies new loci associated with longitudinal blood pressure traits
Source: Sci Rep. 2021 Feb 18;11:4075. doi: 10.1038/s41598-021-83450-3 (PMC7893038; doi:10.1038/s41598-021-83450-3)
Supplement: Supplementary file 1 — Supplementary Information 1. [file 41598_2021_83450_MOESM1_ESM.pdf]

Trans-ethnic meta-analysis identifies new loci associated with longitudinal blood pressure traits

Mateus H. Gouveia[1], Amy R. Bentley[1], Hampton Leonard [2,3], Karlijn A.C. Meeks[1], Kenneth Ekoru[1], Guanjie Chen[1], Michael A. Nalls[2,3], Eleanor M. Simonsick [4], Eduardo Tarazona-Santos[5], Maria Fernanda Lima-Costa[6], Adebowale Adeyemo[1], Daniel Shriner[1*], and Charles N. Rotimi[1*]

1. Center for Research on Genomics and Global Health, National Human Genome Research Institute, Bethesda, MD, 20892, USA;
2. Laboratory of Neurogenetics, National Institute on Aging, National Institutes of Health, Bethesda, MD, 20892, USA;
3. Data Tecnica International, Glen Echo, MD, 20812, USA;
4. Longitudinal Studies Section, Translational Gerontology Branch, National Institute on Aging, Baltimore, Maryland;
5. Departamento de Genética, Ecologia e Evolução, Instituto de Ciências Biológicas, Universidade Federal de Minas Gerais, Belo Horizonte, MG, 31270-901, Brazil;
6. Instituto de Pesquisa Rene Rachou, Fundação Oswaldo Cruz, Belo Horizonte, MG, 30190-002, Brazil.

Supplementary Information (SI)

SI Text

Using longitudinal data, we identified 10 BP loci (Fig. 1, and Figs. S3, S5, S6, S8, S9, Table 2 and Tables S1-S4,): six in African Americans (AA), three in European Americans (EA), and one in Chinese and Hispanic American individuals. The meta-analysis of cross-sectional BP identified nine loci (Fig. 1, Figs. S3, S7, S10, Table 2 and Tables S1-S4): five in African Americans (AA), two in Hispanics, one in Brazilians, and one in trans-ethnic groups. Here we describe the most promising variants based on our variant- and gene-level annotation (Table S4).

The signal with the smallest *p*-value was the intronic rs140659580-C allele associated with a higher average systolic BP (β = 16.79 mm Hg, SE = 2.62, $p = 7.36\times10^{-10}$) in Hispanic and Chinese American individuals from the MESA study (Figs. S3D, S4 and Tables S1-S4). The rs140659580-C allele frequency is 2% in the Admixed American (AMR) and 3% in the East Asian (EAS) populations from the 1000 Genomes Project (1KGP), but this variant is not observed in other 1KGP populations (Table S1). This variant was also associated ($p = 7.36\times10^{-10}$) with baseline systolic BP (β = 19.05 mm Hg, SE = 3.09). rs140659580 is located in the gene *GLG1* (golgi glycoprotein 1), which is highly expressed in the aorta and coronary arteries and was previously associated with varicose veins [1]. Moreover, pathway analyses mapped *GLG1* to cell surface interactions at the vascular wall and hemostasis pathways [2]. The T allele of the intronic variant rs73601659 was associated with increasing SBP trajectory in AA (Fig. S3A, and Tables S1-S4). This variant is in *CHD9* (Chromodomain Helicase DNA Binding Protein 9), which has been previously associated with

baseline plasma renin activity [3] and plasma fibrinogen concentration [4], which are established predictors of hypertension.

The C allele of variant chr19:46803029 was associated with a higher average systolic BP (β = 16.11 mm Hg, SE = 2.78) only in AA (Tables S1-S4). The 19:46803029 variant is rare (not present in any 1KGP populations) and was observed in only one of our AA studies (MESA). This variant is annotated to gene *HIF3A,* which is associated with enlargement of the right ventricle of the heart in mice [5] and involved in the pro-oxidative response to long-term renal injury in rats [6]. Additionally, *HIF3A* plays an important role in the development of adipose tissue dysfunction in obesity [7] and is an established lipids locus [8].

The rs7248651-C allele was associated with lower systolic BP (β = -2.32, SE = 0.42) in all AA populations. rs7248651 is a statistically significant *eQTL* ($p = 8.19\times10^{-6}$) for the gene *SLC25A42* (Solute Carrier Family 25 Member 42), which plays a critical role in the transport of molecules across the inner mitochondrial membrane. Previous reports showed a link between an *SLC25A42* variant and mitochondrial myopathy [9] and an association between a variant close to *SLC25A42* with SBP [10]. Also, rs7248651 is a statistically significant *eQTL* ($p = 6.62\times10^{-8}$) in the heart atrial appendage for *TMEM161A*, which has a role in protection against oxidative stress and has been associated with lipids [11].

The rs77619464-G allele was associated with lower systolic BP (β = -7.17 mm Hg, SE = 1.29) in all AA studies (Fig. S4A and Tables S1-S4). The rs77619464-G allele is an African-ancestry specific

intronic variant in the gene *ASAP1* (ADP-ribosylation factor (ARF) GTPase-activating protein). *ASAP1* may be involved in the regulation of membrane trafficking and cytoskeleton remodeling [12] and is ubiquitously expressed, including all sampled regions of the heart.

SI Figures

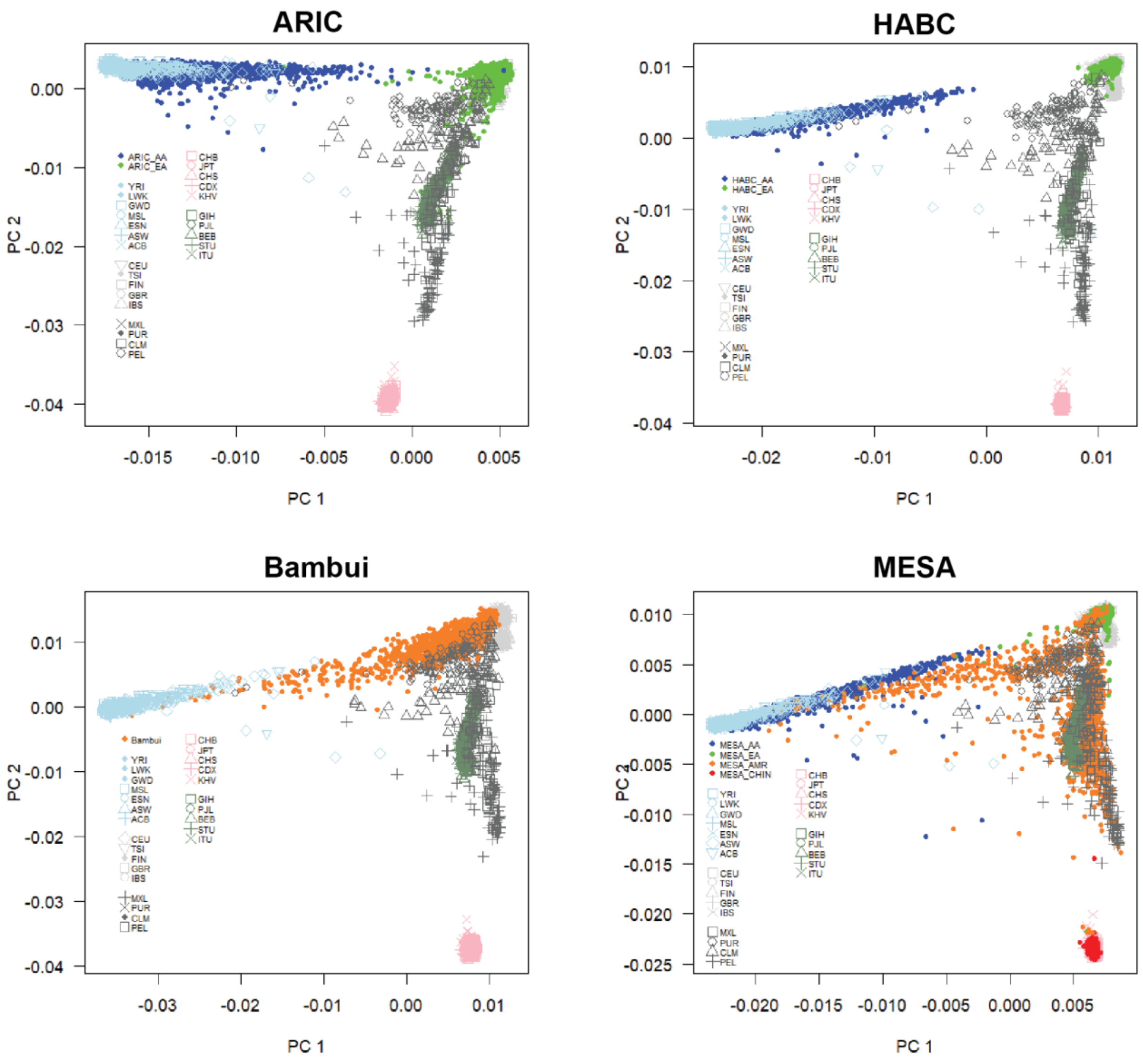

Figure S1. Principal Component Analysis (PCA). Each panel represents the projection in the first two dimensions of the studied populations against the 1000 Genomes Project populations. YRI - Yoruba in Ibadan, Nigeria; LWK - Luhya in Webuye, Kenya; GWD - Gambian in Western Divisions in the Gambia; MSL - Mende in Sierra Leone; ESN - Esan in Nigeria; ASW - Americans of African Ancestry in SW USA; ACB - African Caribbeans in Barbados; CEU - Utah Residents (CEPH) with Northern and Western European Ancestry; TSI - Toscani in Italia; FIN - Finnish in Finland; GBR -

British in England and Scotland; IBS - Iberian Population in Spain; MXL - Mexican Ancestry from Los Angeles USA; PUR - Puerto Ricans from Puerto Rico; CLM - Colombians from Medellin, Colombia; PEL - Peruvians from Lima, Peru; CHB - Han Chinese in Beijing, China; JPT - Japanese in Tokyo, Japan; CHS - Southern Han Chinese; CDX - Chinese Dai in Xishuangbanna, China; KHV - Kinh in Ho Chi Minh City, Vietnam; GIH - Gujarati Indian from Houston, Texas; PJL - Punjabi from Lahore, Pakistan; BEB - Bengali from Bangladesh; STU - Sri Lankan Tamil from the UK; ITU - Indian Telugu from the UK.

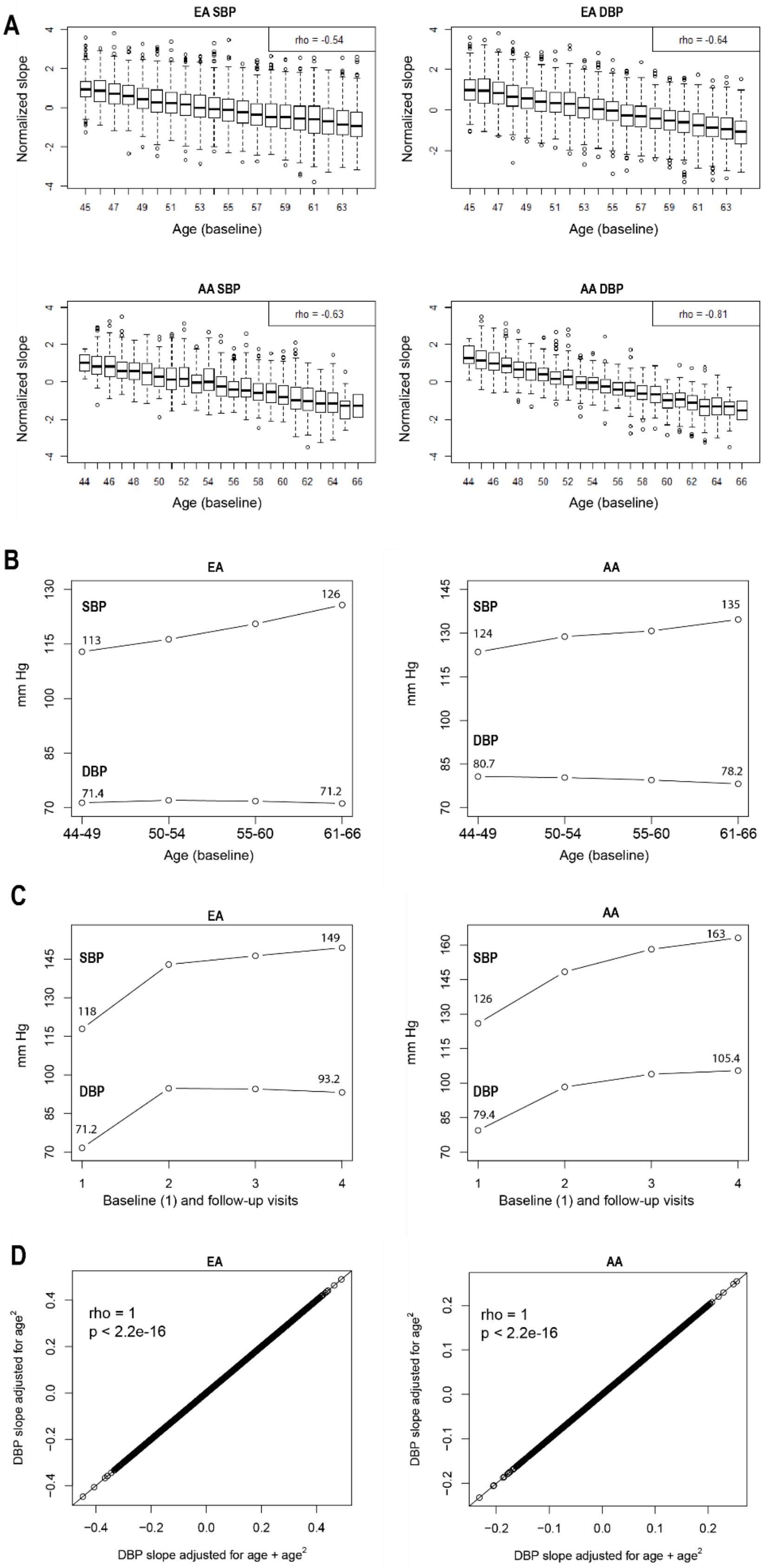
A
EA SBP
rho = -0.54
EA DBP
rho = -0.64
AA SBP
rho = -0.63
AA DBP
rho = -0.81
Normalized slope
Age (baseline)
B
EA
AA
SBP
DBP
113
126
71.4
71.2
124
135
80.7
78.2
mm Hg
44-49
50-54
55-60
61-66
Age (baseline)
C
EA
AA
118
149
71.2
93.2
126
163
79.4
105.4
Baseline (1) and follow-up visits
D
EA
AA
rho = 1
p < 2.2e-16
DBP slope adjusted for age²
DBP slope adjusted for age + age²

Figure S2. BP trajectory and sensitivity analysis. A) Distribution of the individual's slope of systolic and diastolic blood pressure in relation to baseline age; B) Mean systolic and diastolic blood pressures by age; C) Mean systolic and diastolic blood pressures by visit; and D) Sensitivity analysis comparing the estimates of the individual's DBP slope adjusted for age^2 vs. age + age^2. The data shown are from 9,170 European Americans (EA) and 2,764 African Americans (AA) from the ARIC study.

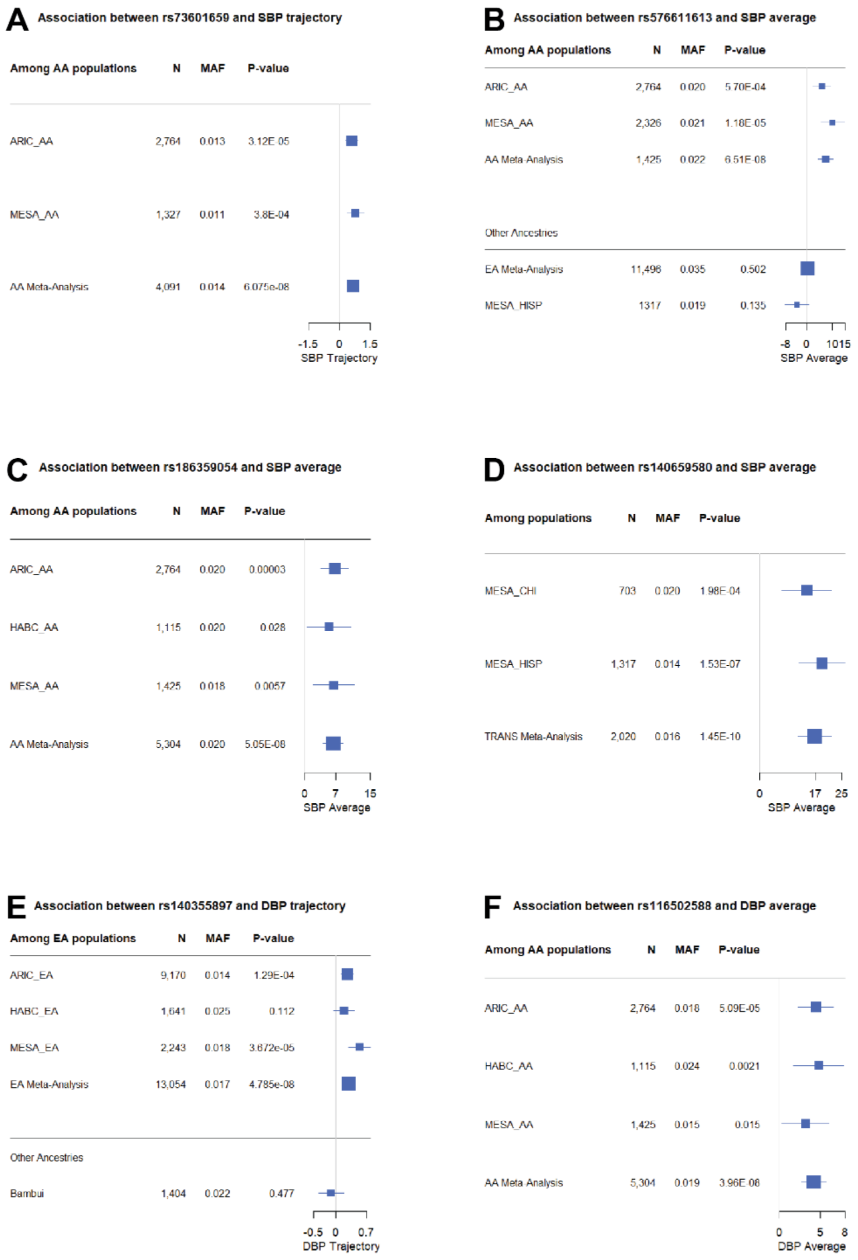

Figure S3. Forest plots of the genome-wide statistically **significant associations of BP longitudinal data.** Forest plots show β values (95% confidence intervals) and *P*-values from the linear regression of longitudinal outcomes (BP trajectory or BP average) adjusted by age^2, sex, BMI, principal components (PC1 and PC2), and the use of antihypertensive medications. Populations not shown in the plots did not have the genetic variant to perform association

analyses due to low allele frequency or because the variant was pruned during quality control.

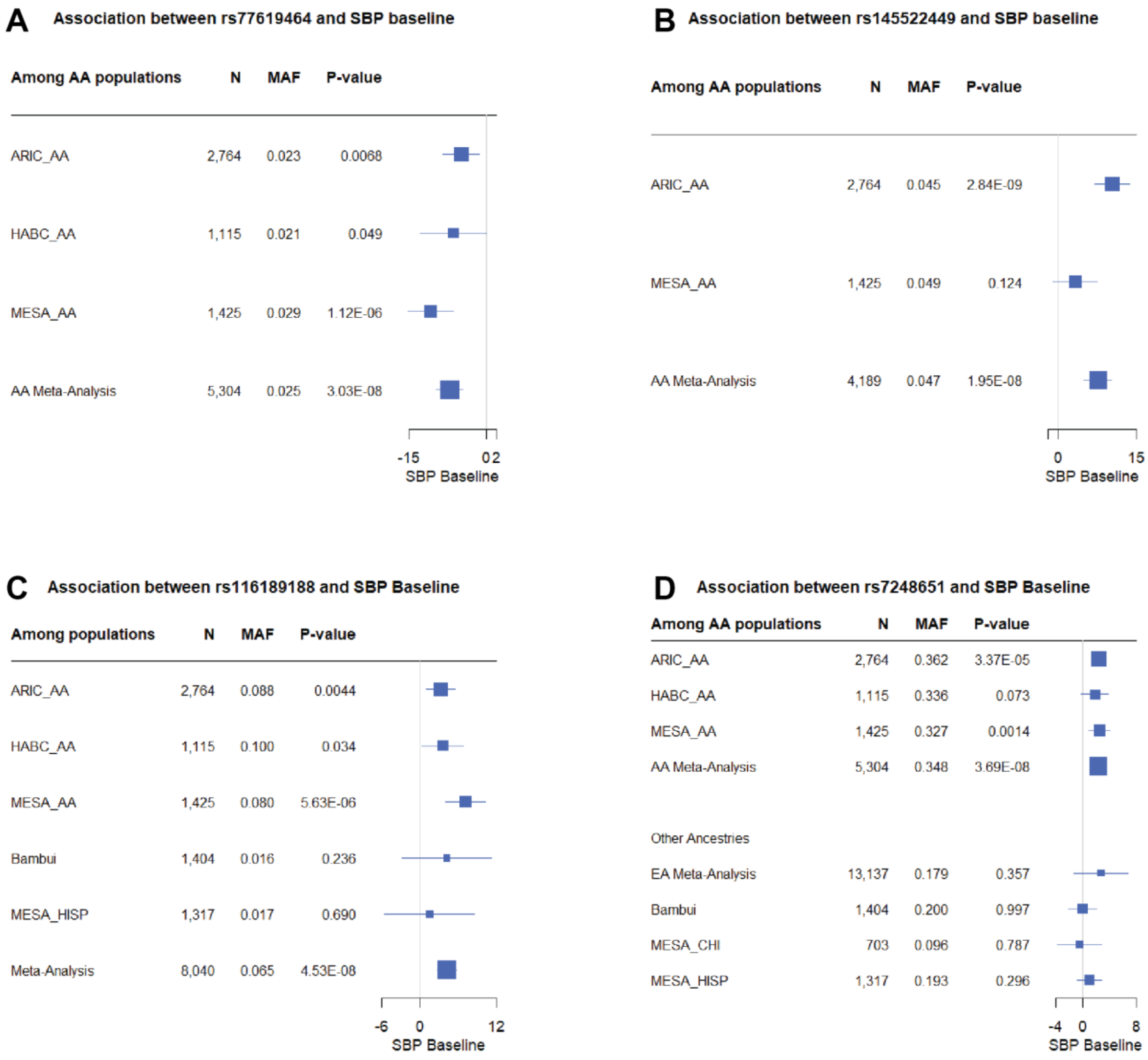

Figure S4. Forest plots of the genome-wide statistically **significant associations of BP baseline data.** Forest plots show β values (95% confidence intervals) and *P*-values from the linear regression of longitudinal outcomes (BP trajectory or BP average) adjusted by age^2, sex, BMI, principal components (PC1 and PC2), and the use of antihypertensive medications. Populations not shown in the plots did not have the genetic variant to perform association analyses due to

low allele frequency or because the variant was pruned during quality control.

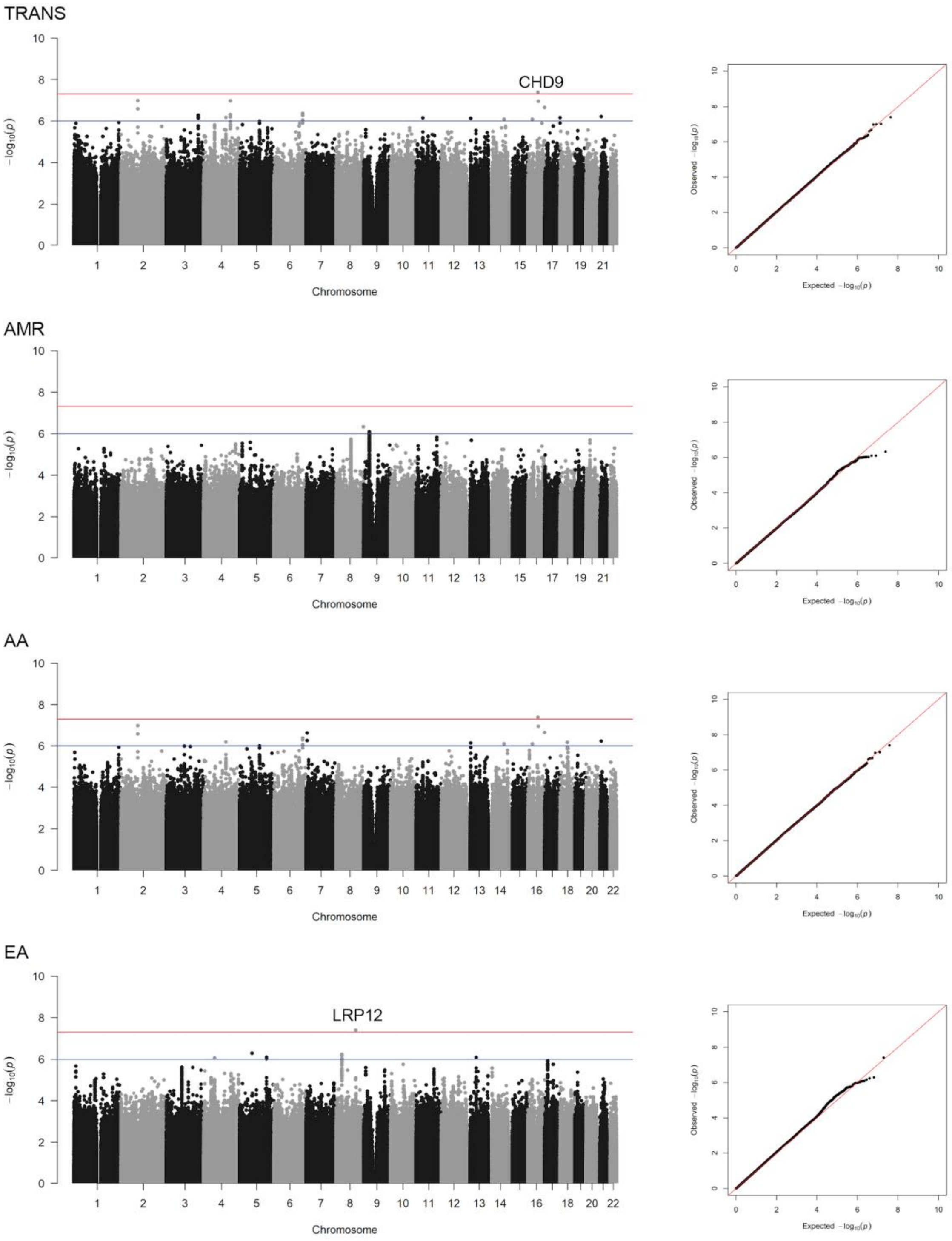

Figure S5. Manhattan and *q-q* plots of the meta-analysis of the trajectory of systolic blood

pressure (SBP). TRANS = the trans-ethnic meta-analysis including individuals from all ancestry groups; AMR = meta-analysis of admixed American individuals (Bambui-Brazil and Latinos from MESA); AA = meta-analysis of African American individuals from ARIC, HABC, and MESA; EA = meta-analysis of European American individuals from ARIC, HABC, and MESA.

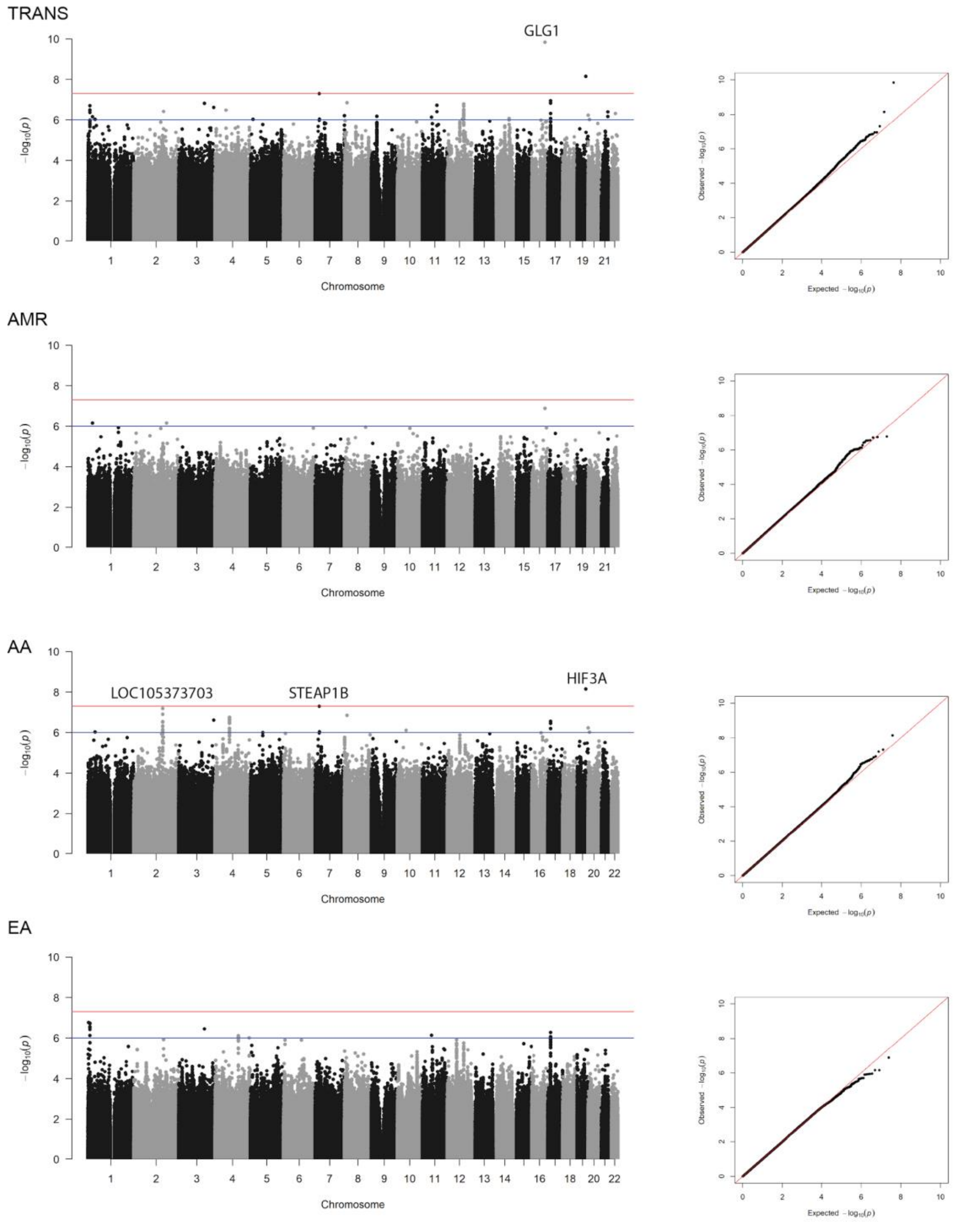

Figure S6. Manhattan and *q-q* plots of the meta-analysis of the average systolic blood pressure (SBP) over time. TRANS = the trans-ethnic meta-analysis including individuals from all ancestry groups; AMR = meta-analysis of admixed American individuals (Bambui-Brazil and Latinos from MESA); AA = meta-analysis of African American individuals from ARIC, HABC, and

MESA; EA = meta-analysis of European American individuals from ARIC, HABC, and MESA.

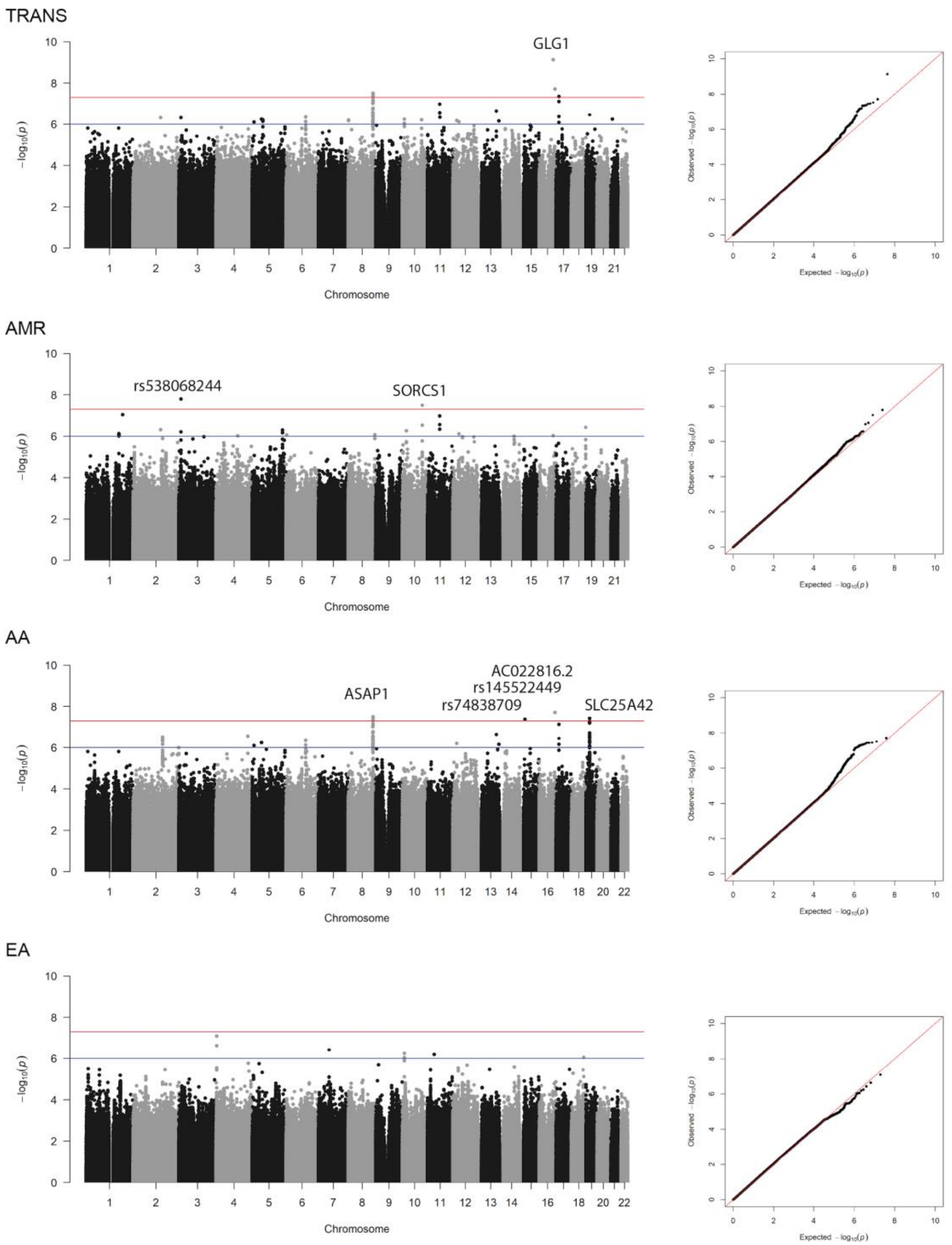

Figure S7. Manhattan and *q-q* plots of the meta-analysis of the baseline systolic blood pressure (SBP). TRANS = the trans-ethnic meta-analysis including individuals from all ancestry groups; AMR = meta-analysis of admixed American individuals (Bambui-Brazil and Latinos from MESA); AA = meta-analysis of African American individuals from ARIC, HABC, and MESA; EA = meta-analysis of European American individuals from ARIC, HABC, and MESA.

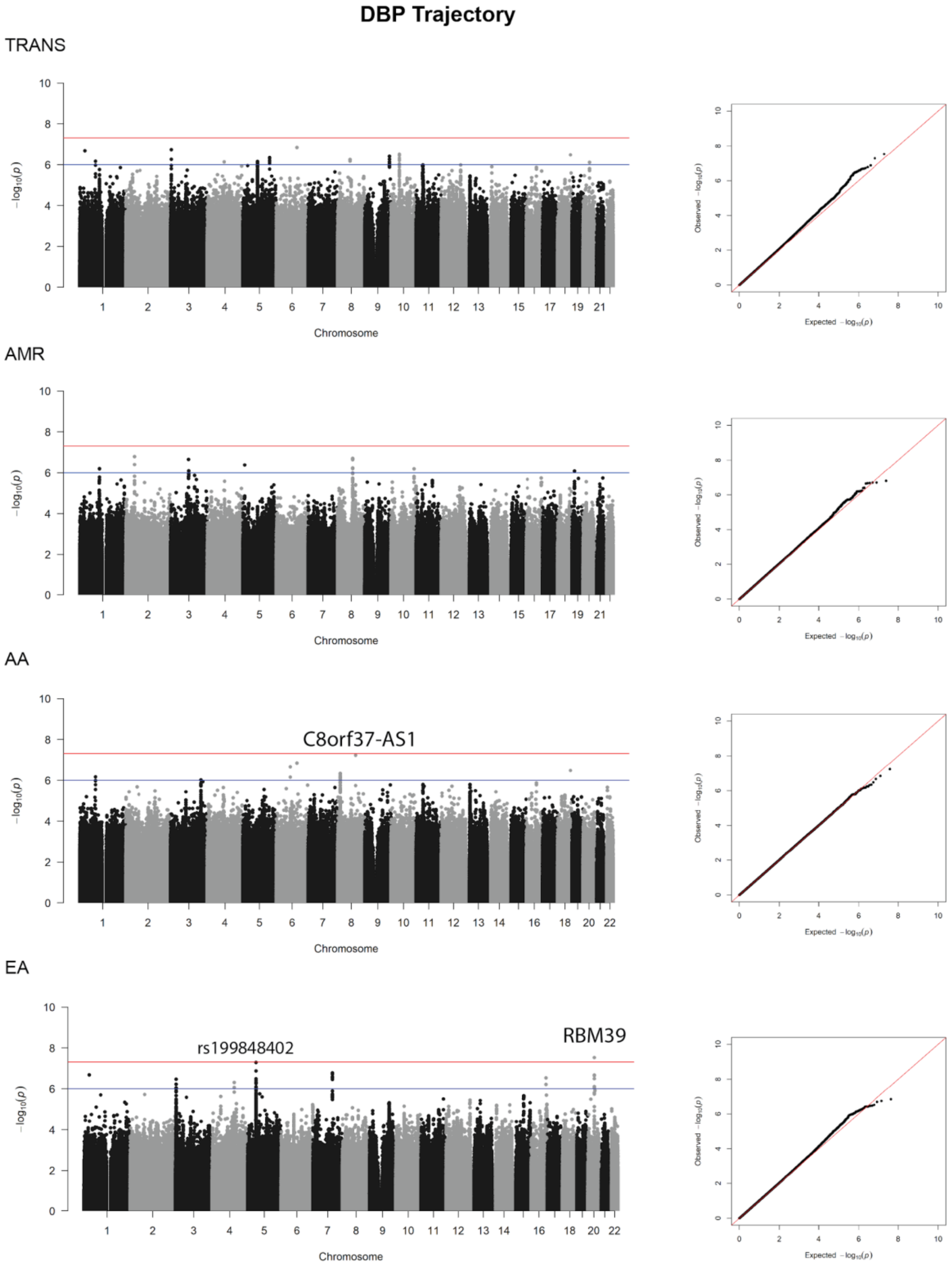

Figure S8. Manhattan and *q-q* plots of the meta-analysis of the trajectory of the diastolic blood pressure (DBP). TRANS = the trans-ethnic meta-analysis including individuals from all

ancestry groups; AMR = meta-analysis of admixed American individuals (Bambui-Brazil and Latinos from MESA); AA = meta-analysis of African American individuals from ARIC, HABC, and MESA; EA = meta-analysis of European American individuals from ARIC, HABC, and MESA.

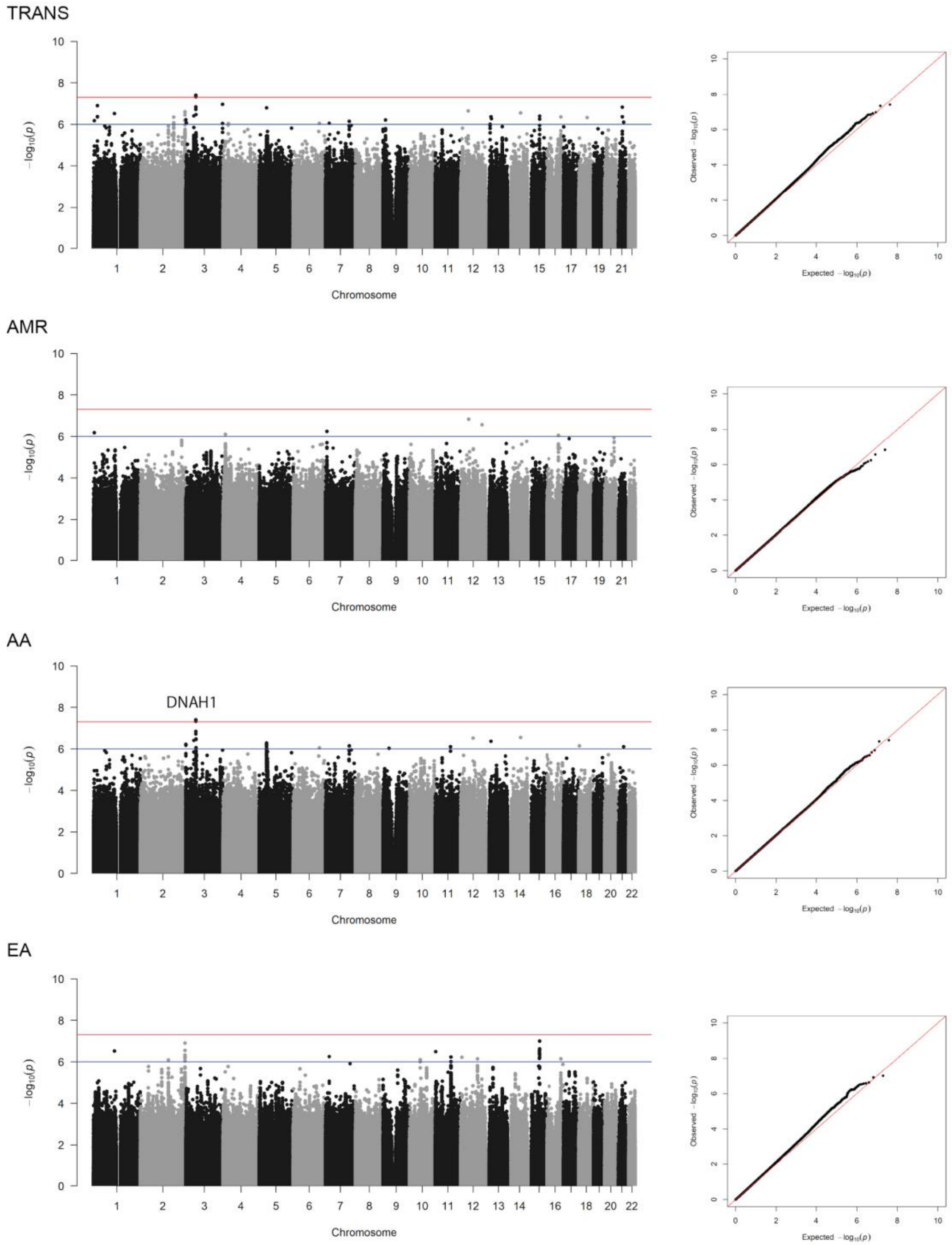

Figure S9. Manhattan and *q-q* plots of the meta-analysis of the average diastolic blood pressure (SBP) over time. TRANS = the trans-ethnic meta-analysis including individuals from all ancestry groups; AMR = meta-analysis of admixed American individuals (Bambui-Brazil and Latinos from MESA); AA = meta-analysis of African American individuals from ARIC, HABC, and MESA; EA = meta-analysis of European American individuals from ARIC, HABC, and MESA.

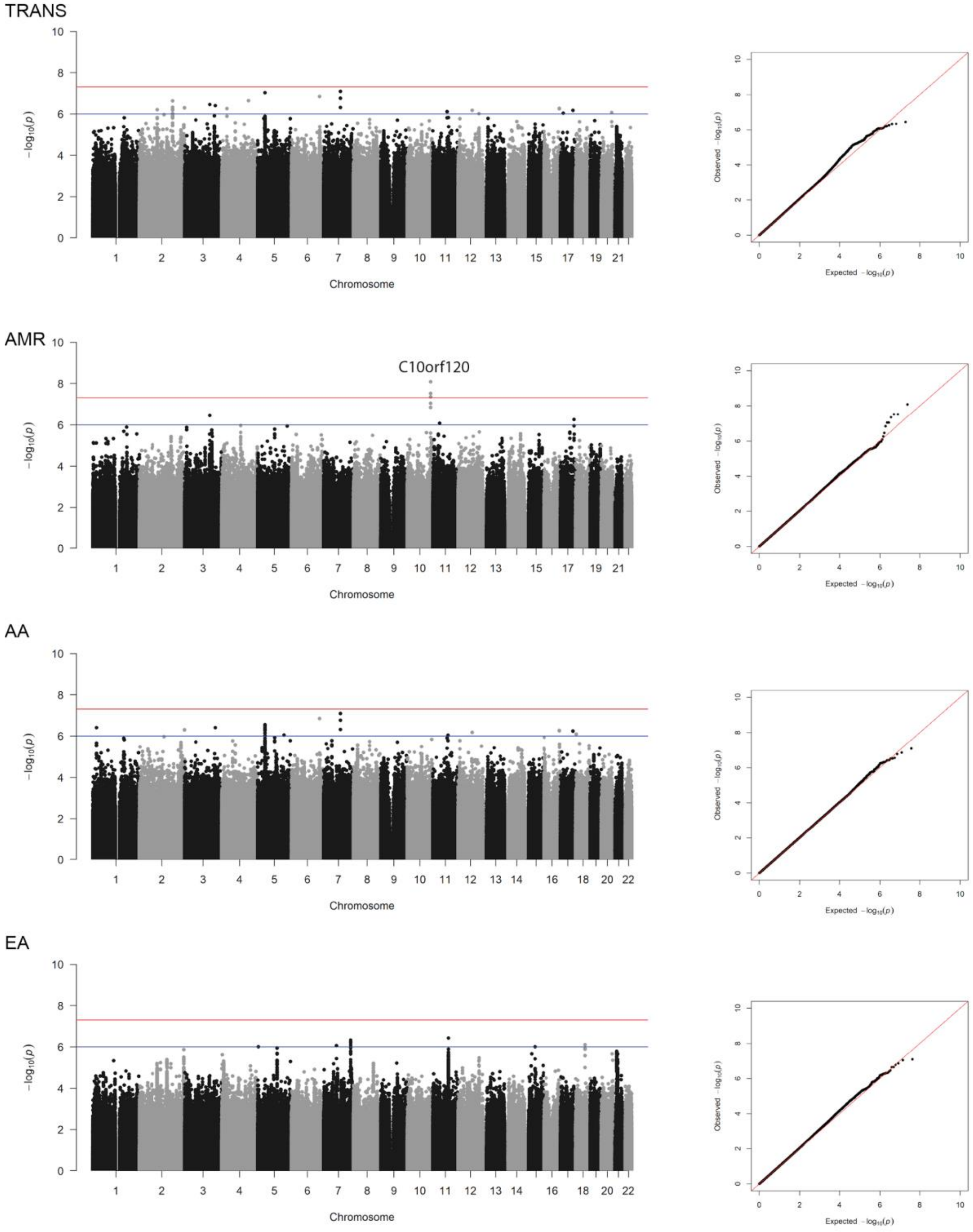

Figure S10. Manhattan and *q-q* plots of the meta-analysis of the baseline diastolic blood pressure (SBP) over time. TRANS = the trans-ethnic meta-analysis including individuals from all ancestry groups; AMR = meta-analysis of admixed American individuals (Bambui-Brazil and

Latinos from MESA); AA = meta-analysis of African American individuals from ARIC, HABC, and MESA; EA = meta-analysis of European American individuals from ARIC, HABC, and MESA.

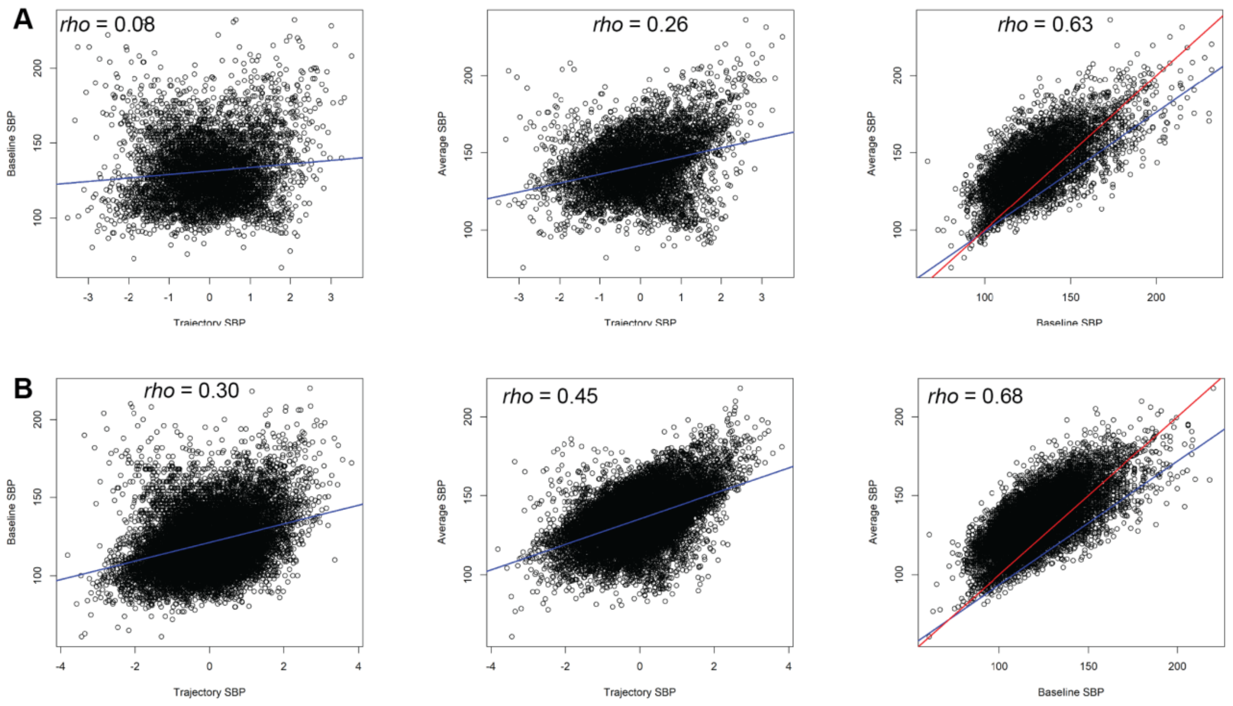

Figure S11. Comparison between longitudinal and cross-sectional SBP data. Correlation between longitudinal and baseline SBP traits using (A) African American and (B) European American data. We combined 5,293 African Americans and 13,120 European Americans from the ARIC, MESA, and HABC studies (Table 1). For reference, the diagonal red line indicates rho = 1, that is, perfect positive correlation.

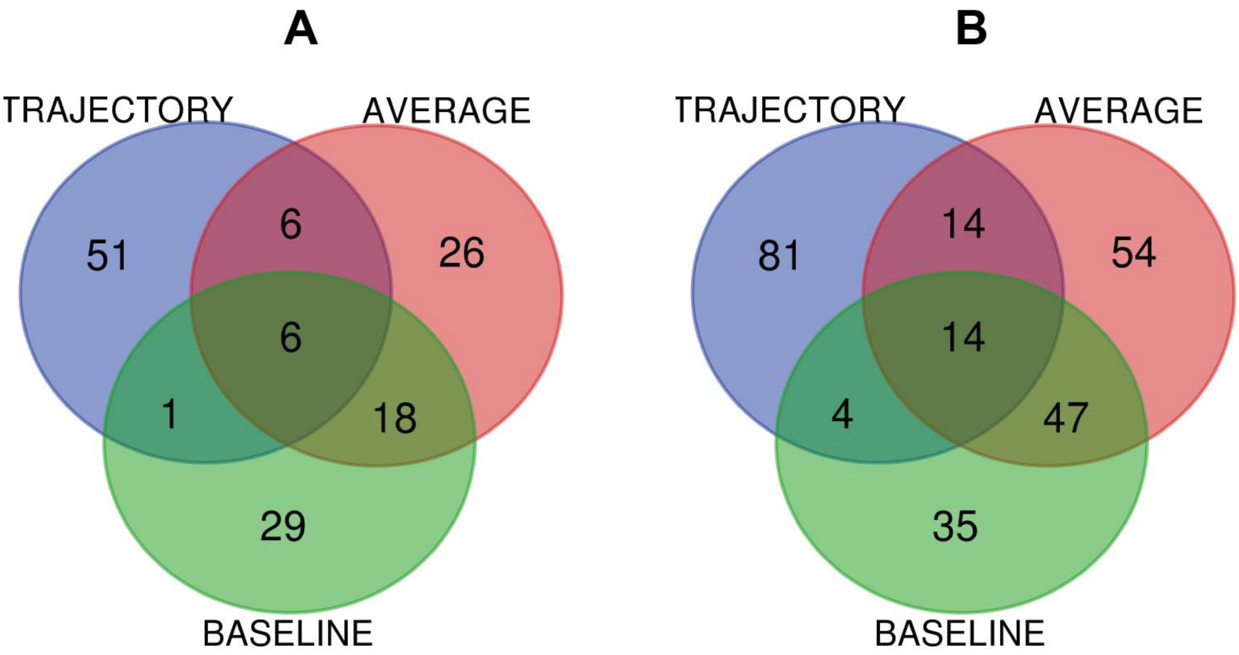

Figure S12. Number of GWAS Catalog hits identified by our meta-analysis discovery data.

Replications using A) systolic and B) diastolic blood pressure meta-analysis using the longitudinal (trajectory and average) and baseline outcomes.

References

1. Fukaya, E. *et al.* Clinical and Genetic Determinants of Varicose Veins: Prospective, Community-Based Study of≈ 500 000 Individuals. *Circulation* **138**, 2869–2880 (2018).
2. Mi, H. *et al.* PANTHER version 11: expanded annotation data from Gene Ontology and Reactome pathways, and data analysis tool enhancements. *Nucleic Acids Res.* **45**, D183–D189 (2017).
3. McDonough, C. W. *et al.* Genetic Variants Influencing Plasma Renin Activity in Hypertensive Patients From the PEAR Study (Pharmacogenomic Evaluation of Antihypertensive Responses). *Circ Genom Precis Med* **11**, e001854 (2018).

4. Sabater-Lleal, M. *et al.* Multiethnic meta-analysis of genome-wide association studies in >100 000 subjects identifies 23 fibrinogen-associated Loci but no strong evidence of a causal association between circulating fibrinogen and cardiovascular disease. *Circulation* **128**, 1310–1324 (2013).

5. Yamashita, T. *et al.* Abnormal heart development and lung remodeling in mice lacking the hypoxia-inducible factor-related basic helix-loop-helix PAS protein NEPAS. *Mol. Cell. Biol.* **28**, 1285–1297 (2008).

6. Wesseling, S. *et al.* Transcriptome-based identification of pro- and antioxidative gene expression in kidney cortex of nitric oxide-depleted rats. *Physiological Genomics* vol. 28 158–167 (2007).

7. Pfeiffer, S. *et al.* Hypoxia-inducible factor 3A gene expression and methylation in adipose tissue is related to adipose tissue dysfunction. *Sci. Rep.* **6**, 27969 (2016).

8. Buniello, A. *et al.* The NHGRI-EBI GWAS Catalog of published genome-wide association studies, targeted arrays and summary statistics 2019. *Nucleic Acids Res.* **47**, D1005–D1012 (2019).

9. Shamseldin, H. E. *et al.* Mutation of the mitochondrial carrier SLC25A42 causes a novel form of mitochondrial myopathy in humans. *Hum. Genet.* **135**, 21–30 (2016).

10. Fox, E. R. *et al.* Association of genetic variation with systolic and diastolic blood pressure among African Americans: the Candidate Gene Association Resource study. *Hum. Mol. Genet.* **20**, 2273–2284 (2011).

11. Kettunen, J. *et al.* Genome-wide study for circulating metabolites identifies 62 loci and reveals novel systemic effects of LPA. Nat Commun. 2016; 7: 11122.

12. Brown, M. T. *et al.* ASAP1, a phospholipid-dependent arf GTPase-activating protein that associates with and is phosphorylated by Src. *Mol. Cell. Biol.* **18**, 7038–7051 (1998).
